# Molecular Pathways of Breast Cancer in Systemic Sclerosis: Exploratory Immunohistochemical Analysis from the Sclero-Breast Study

**DOI:** 10.3390/jpm12122007

**Published:** 2022-12-03

**Authors:** Chrystel Isca, Amelia Spinella, Angela Toss, Marco de Pinto, Guido Ficarra, Luca Fabbiani, Anna Iannone, Luca Magnani, Federica Lumetti, Pierluca Macripò, Caterina Vacchi, Elisa Gasparini, Simonetta Piana, Laura Cortesi, Antonino Maiorana, Carlo Salvarani, Massimo Dominici, Dilia Giuggioli

**Affiliations:** 1 Department of Oncology and Hematology, University Hospital of Modena, 41124 Modena, Italy; 2 SSc Unit, Rheumatology Unit, University of Modena and Reggio Emilia, 41124 Modena, Italy; 3 Department of Medical and Surgical Sciences for Children and Adults, University of Modena and Reggio Emilia, 41124 Modena, Italy; 4 Pathology Unit, University Hospital of Modena, 41124 Modena, Italy; 5 Department of Surgery, Medicine, Dentistry and Morphological Sciences with Transplant Surgery, Oncology and Regenerative Medicine Relevance, University of Modena and Reggio Emilia, 41124 Modena, Italy; 6 Rheumatology Unit, IRCCS Santa Maria Nuova Reggio Emilia, 42123 Reggio Emilia, Italy; 7 Department of Oncology, Arcispedale S. Maria Nuova IRCCS, 42123 Reggio Emilia, Italy; 8 Pathology Unit, Azienda USL Reggio Emilia, IRCCS, 42123 Reggio Emilia, Italy

**Keywords:** breast cancer, systemic sclerosis, immunohistochemistry, pathways, TILs, PD-L1, cancer biomarkers, de-escalation therapy

## Abstract

Several authors reported an increased risk of cancer in SSc patients, including breast cancer (BC). Nevertheless, the mechanisms underlying this association have not yet been clarified. SSc and BC share several molecular pathways, which seem to play a common etiopathogenetic role. The previously published Sclero-Breast study demonstrated the development of BC with a good prognosis among these patients, which could be explained by an autoimmune background as a possible mechanism for limiting tumor extension. Here, we report the results of an IHC analysis of molecular pathways known to be common drivers for both diseases, with the aim to better define the mechanisms underlying a good prognosis of BC in patients affected by SSc. The analysis demonstrated higher TILs rates in all BC subgroups, with a high rate of PD-L1 expression especially in TNBC and HER2-positive BC, suggesting a less aggressive behavior in these patients compared to the general population. These results support a possible de-escalation strategy of cancer therapies in these fragile patients. These data could represent a starting point for future prospective studies based on the clinical application of these biomarkers with a larger sample size to promote a personalized and targeted oncological treatment for this specific subset of patients.

## 1. Introduction

Systemic Sclerosis (SSc) is a rare connective tissue disease characterized by vasculopathy and fibrosis of the skin and internal organs [1,2,3]. Several authors reported an increased risk of cancer (1,5 fold) in SSc patients compared to general population, especially lung cancer, haematological malignancies and breast cancer (BC) [4,5,6,7,8,9]. Nevertheless, to our knowledge, the mechanisms underlying this association have not been well clarified. Many theories have been proposed: the exposure to immunosuppressive therapies, a genetic predisposition to both pathologies, epigenetic changes, environmental factors, and paraneoplastic syndrome [10,11,12,13,14].

Considering the known close relationship between BC and SSc [7,15,16], in our recently published study [17] focused on the analysis of clinical-pathological features of breast cancer in patients with systemic sclerosis, we observed the development of BC with early stages and good prognosis among these patients. The results of this work firstly suggested the role of the autoimmune background, which characterizes SSc as a possible mechanism for limiting tumor extension, and secondly as a possible de-escalation strategy of cancer therapies for these patients with multimorbidity.

The present multicenter retrospective study, performed at Modena University Hospital and Reggio Emilia Hospital, explored the immunohistochemical (IHC) expression of potential biomarkers involved in the molecular pathways at the basis of SSc and BC etiopathogenesis. In detail, PI3K, mTOR, TGFβ, PDGFRα, PDGFRβ, VEGF, EGFR, IL 6, CTLA-4, PD-L1 and TILs were explored

## 2. Materials and Methods

The Sclero-Breast study was an observational multicenter retrospective study performed at Modena University Hospital and Reggio Emilia Hospital in Northern Italy. Thirty-three women affected by SSc [18] with a personal history of BC were enrolled between January 2017 and December 2019 with the aim to evaluate the clinical and pathological features of BC developed by SSc patients. Clinical and pathological characteristics of BC and clinical-rheumatological features related to SSc were also analyzed [17].

For twenty-two patients enrolled in the Sclero-Breast study, BC tissues were available in our archives. Formalin-fixed, paraffin-embedded block from biopsies or surgical samples were used. An IHC method was performed on 5-micron thickness sections of the paraffin-blocks. The device used for the immunohistochemical staining was Ventana BenchMark XT^®^, which, thanks to a “bar code” system, could recognize both the reagents and the slides, by automatically performing all the steps of a staining protocol, specific for each antibody used. For chromogenic detection, we used a XT ultraView DAB kit.

IHC analysis was performed using specific antibodies to evaluate biomarkers and pathways involved in BC and SSc development. In detail, the antibodies used included PI3K (p85α) (Abcam), mTOR (Abcam), TGFβ (Biorad), PDGFRα (Santa Cruz), PDGFRβ (Santa Cruz), VEGF (Thermo Fisher), EGFR (Santa Cruz), IL-6 (Gp130) (Santa Cruz), CTLA-4 (Abcam), and PD-L1(sp142) (Ventana). In addition, we reported TILs percentage (stromal tumor-infiltrating lymphocytes) of each sample. The following scores were assigned for general IHC analysis: (-) negative, (1+) positive tumor cells < 20%, (2+) positive tumor cells 20–50%, and (3+) positive tumor cells >50%. For PD-L1 evaluation, we considered a positivity in the case of PD-L1 expression ≥1% in infiltrating inflammatory cells. For TILs quantification, we applied the following score: 0 (negative), ≤50% (low-median expression), >50% (high expression). Correlations between rheumatological [17] and oncological features were also performed.

Statistical analysis was performed using SPSS (IBM software, New York, NY, USA, version 26.0). Fisher’s exact test was applied. The 95% confidence interval and Odds Ratio were also estimated for results showing trend towards significance. *p* < 0.05 was considered statistically significant.

Approval for this study was obtained from the local ‘Area Vasta Emilia Nord (AVEN)’ Ethics Committee (practice n.376/17, protocol n.4442/EC) and conducted in accordance with the Helsinki Declaration. All patients released a written informed consent.

## 3. Results

### 3.1. Pathological Features of Breast Cancer in the Study Population

Overall, 33 women affected by SSc with a personal history of BC were enrolled in the Sclero-Breast study. Median age at SSc onset was 61.36 ± 9.24 SD years (range 45–75). For 22 of these patients, BC tissues were available in our Pathology Units. Clinical and pathological features of the 22 patients with available BC samples are reported in Table 1. In brief, the median age at diagnosis was 61 years old. Most patients received a diagnosis of invasive ductal carcinoma (75%), followed by lobular invasive carcinoma (20%) and a tubular histotype (5%). Only two patients received a diagnosis of ductal carcinoma in situ. Considering molecular subtype, 60% of BC showed a Luminal-like phenotype, with equal representation of HER2 positive and triple negative subtypes (20%, respectively). A low Mib 1 expression (≤20%) was most prevalent. BC disease was mainly characterized by low clinical and pathological stages (64% and 27% of patients with a clinical stage I-II, 54% and 23% of cases with pathological stage I-II).

**Figure 1:**
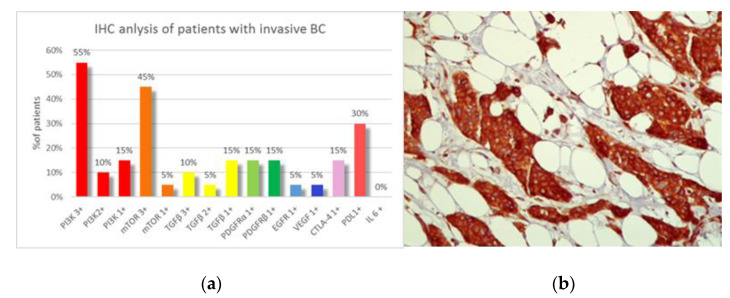
(**a**): Immunohistochemical analysis of samples of patients with invasive BC (20 patients). (**b**): Histological sample of BC with PI3K over-expression (score 3+).

**Figure 2:**
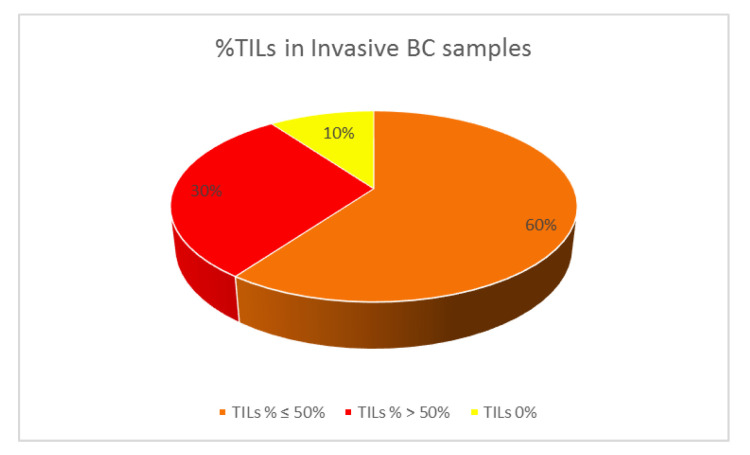
TILs% expression in histological samples of patients with invasive BC (20 patients).

The first IHC analysis was performed on the samples of invasive BC patients (20 patients) (Figure 1a) and showed a prevalence of high PI3K expression (score of 3+ in 55% of cases) (Figure 1a,b) with mTOR over-expression in 45% of cases. TGFβ was positive in 30% of cases, with a prevalence of a score 1+. PDGFRα, PDGFRβ, EGFR, VEGF, and CTLA-4 expression was poorly represented (Figure 1a). IL-6 was negative in all patients. A PD-L1 positivity was detected in 30% of cases, with high TILs expression in 30% of samples (Figure 2). Histological samples from the two patients affected by ductal carcinoma in situ were characterized by a negativity of almost all parameters analyzed, with the exception of a low expression of TGFβ (score 1+) in one patient and a low mTOR expression (score 1+) in another. A medium-high TILs expression was reported (40% and 90%, respectively).

**Figure 3:**
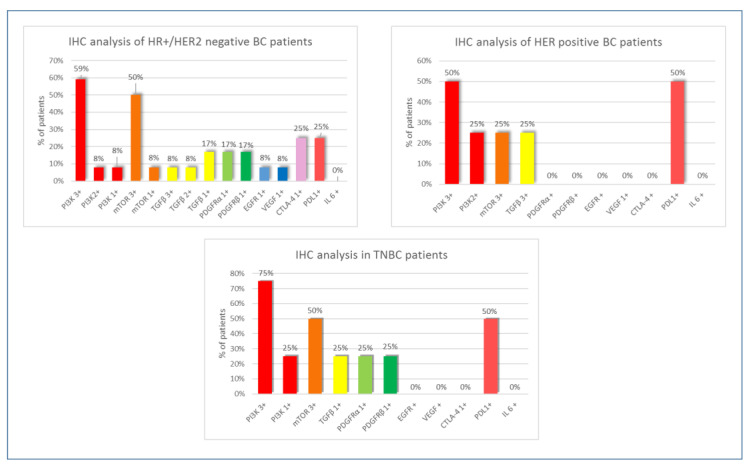
Immunohistochemical analysis of Hormone positive/HER2 negative BC patients, HER2 positive BC patients and Triple Negative BC patients.

IHC analysis was also performed according to BC subtypes. The first group was represented by Hormone positive/HER2 (HR+/HER2) negative breast cancer patients (12 women), the second by HER2 positive BC patients (4 women) and finally the third by Triple negative BC (TNBC) patients (4 women). As reported in Figure 3, the group of HR+/HER2 negative BC patients showed a high PI3K expression (score 3+) in most cases (59%) with an mTOR over-expression in half of the cases. TGFβ was positive in 33% of samples (mostly with a score 1+), while a mild expression of other parameters was detected (17% with a score +). CTLA-4 and PD-L1 were positive in 25% of cases, with high TILs expression in 25% of histological samples. HER2 positive BC patients showed a high PI3K positivity in 50% of cases with mTOR positivity (score 3+) in 25% of samples and high TGFβ expression (score 3+) in 25% (Figure 3). All other parameters were negative. PD-L1 was positive in 50% of cases with high TILs expression in 25% of histological samples. In the Triple Negative BC group, PI3K over-expression was found in 75% of patients with half of cases represented by an mTOR score 3+ (Figure 3). A low positivity for TGFβ, PDGFRα and PDGFRβ (score 1+) in 25% of cases was detected. PD-L1 was positive in 50% of histological samples with high TILs representation (80% of total cell count) in 50% of patients.

### 3.2. Systemic Sclerosis and Breast Cancer Correlations in the Study Population

The rheumatological characteristics of the overall SSc population are listed in the Sclero-Breast study [17]. Correlations between the main rheumatological features and some immunoistochemical aspects of breast cancer were also performed: interesting findings were observed as regards to autoantibody profile (ANA, ACA, ANoA positivity), skin and heart involvement (skin ulcers and PAPs, respectively). The most significant correlations are reported in Table 2.

In detail, considering antibodies pattern of SSc patients, a significant correlation between ANA and PI3K expression (*p* = 0.044) and between ANA and PDGFRβ (*p =* 0.031) was observed, with a trend towards significance regarding to ACA and PDGFRβ (*p =* 0.074) and ANoA and PI3K (*p =* 0.079).

No significant correlations were observed for the other parameters analyzed, but trends toward significance were noticed regarding other autoantibodies, skin ulcers and pulmonary hypertension assessed by PAPs at echo (Table 2).

## 4. Discussion

The Sclero-Breast study was a retrospective observational study that explored the clinical and pathological characteristics of BC in patients affected by SSc. In the first part of the work, which included 33 patients [17], we observed the development of early BC in most of the cases, with a prevalence of clinical stages I-II (93.1% of patients) and only one patient with metastatic disease. In the study, good prognostic features were detected, such as low mib-1 values (70.8% of cases) and Hormone receptor positive tumors (76.1%). According to such good prognostic features, only 10.3% of patients underwent neoadjuvant chemotherapy, and 66.6% breast-conserving surgery. Interstitial lung disease was reported in more than half of patients enrolled and in all the six deceased patients. In all cases, the cause of death was not related to cancer, but to PAH induced by the autoimmune disease [17].

Here, we report the results of an IHC analysis of molecular pathways known to be common drivers for both diseases to better define the mechanisms underlying the good prognosis of BC in patients affected by SSc.

In particular, PI3K and mTOR are known to be involved in SSc dermal fibrosis [19] and have demonstrated an active role in tumor cell proliferation in BC [20]. Both are targets of several approved drugs or therapies under investigation for stage IV BC, such as alpelisib and everolimus [21,22]. Additionally, TGFβ, a factor responsible of fibroblasts activation in SSc [23], is a major regulator of many BC processes, including proliferation, differentiation, migration, immunity, and apoptosis [24]. PDGFRα and PDGFRβ released by endothelial cells to regulate fibroblast activity in SSc [22], have shown a predictive and prognostic role in BC [25,26] and a potential role as treatment targets [27]. VEGF, which is over-expressed in SSc patients [28], is a well-known angiogenetic factor, promoting tumor growth and therefore, a target of Bevacizumab in the metastatic BC treatment. Furthermore, EGFR involved in autoimmune diseases, is a target of Lapatinib, approved for the treatment of HER2-positive metastatic BC [29] and other drugs included in preclinical and clinical trials. IL 6 regulates αSMA expression in myofibroblasts [23] and controls BC cell growth, metastasis, and self-renewal of cancer stem cells [24,30]. Cytotoxic T-Lymphocyte Antigen 4 (CTLA-4) is a target of several immunotherapies such as Tremelimumab and Ipilimumab, already included in clinical trials for BC patients [31]. Finally, Programmed Death Ligand 1 (PD-L1), which demonstrated a prognostic and predictive role in BC [32,33], is an important target of some immunotherapies, such as Atezolizumab, which has been approved for the treatment of PD-L1 positive metastatic triple negative BC [34].

TILs percentage is known to be a strong prognostic factor for early BC, especially in TNBC and HER2 positive BC [35,36]. Indeed, a strong correlation between TILs expression and OS (*p* = 0.003 for intratumoral TILs and *p* = 0.005 for stromal TILs) has been reported, with a hypothetical role in response prediction to Anthracyclines treatment [35]. No standard cut-off was defined in these previous experiences.

Our patients showed higher TILs rate in all BC subgroups compared to women affected by BC without SSc as reported in literature, reaching a percentage of 25% in HR+/HER2 negative BC group (vs. 6% previously reported [36]), 25% in HER2 positive BC group (vs. 16% [36]) and 50% in TNBC group (vs. 20% [36]). Considering the limited number of patients included in our analyses, a larger sample size is certainly needed to confirm these results among different BC subgroups. In case of confirmation, this might be considered as a direct effect of SSc autoimmune activity on BC cells, resulting in tumor growth inhibition.

Several studies have reported the expression status of PD-1/PD-L1 in breast cancer patients, but the results did not reach a consensus. Since breast cancer is highly heterogeneous, PD-1/PD-L1 expression may vary among different molecular subtypes, reaching higher levels in basal like subtypes and lowest in luminal A subtypes [37,38]. Some authors reported a negative prognostic role of high PD-L1 expression [38,39], whereas others demonstrated an association with better metastasis-free and overall specific survival in basal tumors [32].

Our patients reported a high rate of PD-L1 expression especially in the TNBC and HER2 positive BC group (50% each). The early-stage disease of these subgroups in our samples suggests a less aggressive breast cancer compared to the one of women without SSc.

Regarding IHC analysis of PI3K and mTOR expression in our patients affected by invasive BC and SSc, we observed a high-rate expression in overall population (55% and 45%, respectively), but PI3K over-expression was not always associated with a concomitant high mTOR expression. This phenomenon should be elucidated at molecular level to better understand gene expression related to these pathways. Probably, the antibody used for PI3K pathway, that binds the p85α subunit, and the antibody used for mTOR may not be considered as specific markers to detect a real PI3K-mTOR pathways’ activation and should be integrated with other biomarkers. In addition, the good prognosis of our patients in all subgroups is in contrast with a possible PI3K-mTOR pathways’ activation, which are known to be related to endocrine resistance in luminal BC and a worse outcome in HER2 and TNBC [20,40,41,42,43,44]. A study with a larger number of women affected by BC and SSc may help clinicians to verify a possible activation of these pathways, in order to evaluate a targeted treatment based on Everolimus or Alpelisib in patients without interstitial lung disease.

For other biomarkers investigated (IL6, CTLA4, TGFβ, PDGFRα, PDGFR β, EGFR and VEGF) a larger sample size would be required to better verify their reliability as prognostic biomarkers in patients affected by BC and SSc. Moreover, a comparison of these biological characteristics with those of women with breast cancer without systemic sclerosis would also be necessary.

Regarding the analysis of correlations between main rheumatological features and some immunoistochemical aspects of BC, we observed a significant correlation between ANA and PI3K and ANA and PDGFRβ. In detail, BC in ANA positive patients does not express PI3K, while in ANA negative patients PDGFRβ was expressed more frequently.

Furthermore, in SSc patients with increased PAPs we observed a more frequent PI3K and mTOR expression; however, this correlation displayed a trend towards significance (*p =* 0.074 and 0.099, respectively). Finally, BC of patients with skin ulcers reported a positive correlation with mTOR expression, while in patients without skin ulcers, a greater representation of PD-L1 was observed. Both cases showed a trend towards significance (*p =* 0.059 and 0.067, respectively).

The main limitations of this study are certainly its retrospective nature, the lack of a control group represented by women affected by BC without SSc and the small sample size of patients studied. Secondly, for some biomarkers, especially for those with a negative expression, the antibodies used for IHC analysis may not have been sufficiently sensitive to investigate some molecular pathways involved. Furthermore, given the small size of the sample, it is not possible to draw clear clinical conclusions regarding BC and SSc correlations, and these associations should be evaluated in future research. In particular, the exploration for possible correlations with vascular and/or fibrotic processes and with autoimmunity panel enriched with SSc-associated autoantibodies would be desirable, also in consideration of intriguing literature data on this topic [45,46].

Finally, these reported preliminary results and the good prognosis of breast cancer in patients affected by SSc [17], could be correlated with a possible de-escalation strategy of cancer therapies. In particular, a mastectomy or conserving surgery without radiotherapy may be suggested, limiting ionizing radiation and chemotherapy to higher-risk cases. For adjuvant hormone treatment, a non-extended therapy may be proposed.

A clinical prospective study with a larger sample size is needed to better clarify the validity of the method used, to verify the efficacy of de-escalation strategies and to explore these predictive biomarkers in all BC subgroups, in order to improve and promote the personalized cancer management in this fragile group of patients.

## 5. Conclusions

SSc and BC share several molecular pathways that seem to play a critical etiopathogenetic role. Even if SSc affects the minority of BC population, a targeted clinical management in this setting is expected. The Sclero-Breast study, based on the evaluation of clinical and pathological features of BC in patients affected by SSc, demonstrated the development of BC with a good prognosis, which could be related to the autoimmune effect on tumor containment.

The IHC analysis that investigated the potential biomarkers involved in the molecular pathways at the basis of SSc and BC etiophatogenesis, highlighted a high PD-L1 expression associated with high TILs percentage. PI3K and mTOR expression need to be further explored to better clarify the real activation of these pathways among these subjects. No significant expression of other biomarkers was detected.

These preliminary data suggest a possible use of a de-escalation strategy of cancer therapy for these patients. A clinical prospective study would be needed to better clarify the prognostic role of these biomarkers, to introduce them in clinical practice and to verify the efficacy of de-escalation strategy in this setting of fragile patients, always considering their pathognomonic disease features.

## Figures and Tables

**Table 1:** Clinical-pathological features of BC patients affected by SSc with available BC samples. * For pathological stage patients subjected to neoadjuvant treatment were included.

	Number of Patients: 22 pts n°(%)
Median age: 61 years	
Histological examination: DCIS Invasive Carcinoma (Ductal Invasive Carcinoma) (Lobular Invasive Carcinoma) (Tubular Carcinoma)	2 (9%) 20 (91%) 15 (75%) 4 (20%) 1 (5%)
Molecular Subtype of invasive BC: Luminal A-like Luminal B-like/Her 2 neg Luminal B-like/Her 2 pos Her 2 enriched-like Triple Negative	10 (50%) 2 (10%) 3 (15%) 1 (5%) 4 (20%)
Mib 1: Mib 1 ≤ 20% Mib 1 > 20%	16 (80%) 4 (20%)
Clinical Stage: I II III IV Pathological Stage *: 0 (In situ) I II III IV	14 (64%) 6 (27%) 1 (4.5%) 1 (4.5%) 3 (14%) 12 (54%) 5 (23%) 1 (4.5%) 1 (4.5%)

**Table 2:** Significant correlations between main SSc features and some BC immunoistochemical aspects.

	*p* Value	OR (IC 95%)
ANA PI3K PDGFRβ	**0.044** **0.031**	2 (1.185; 3.377) 0.235 (0.100; 0.554)
ACA PDGFRβ	0.074	0.353 (0.185; 0.672)
AnoA PI3K	0.079	0.667 (0.379; 1.174)
PAPs PI3K mTOR	0.074 0.099	8.333 (0.776; 89.470) 4.667 (0.765; 28.466)
Ulcers PD-L1 mTOR	0.067 0.059	0.563 (0.365; 0.867) 9.000 (0.854; 94.899)

## Data Availability

The data that support the findings of this study are available from the corresponding author, C.I., upon reasonable request.
